# Supplementary material for: RgCop-A regularized copula based method for gene selection in single-cell RNA-seq data
Source: PLoS Comput Biol. 2021 Oct 19;17(10):e1009464. doi: 10.1371/journal.pcbi.1009464 (PMC8568278; doi:10.1371/journal.pcbi.1009464)
Supplement: S1 Text — sec-1 describes different competing methods and parameter settings, sec-2 describes description of copula in detail, sec-3 describes a short description of regularization techniques. (PDF) [file pcbi.1009464.s001.pdf]

RgCop-A regularized copula based method for gene selection in single cell rna-seq data

Snehalika Lall[1],, Sumanta Ray[2,*]
Sanghamitra Bandyopadhyay[1, *]
[1]Indian Statistical Institute,Kolkata, West Bengal 700108, India.
[2]Genome Data Science, University of Bielefeld, Germany.

1 A short description of competing methods and parameter settings

Four well known gene selection methods in scRNA-seq data are selected for comparisons: *Gini Clust* [1], *PCA Loading* [2], CV^2 *Index* and *Fano Factor* [3]. *Gini Clust* uses Gini Index in feature selection which is used in [1] for rare cell detection in scRNA-seq data. *PCA Loading* selects feature with principal component analysis, which is very common and widely used in scRNA-seq data analysis. CV^2 *Index* is defined as variance to mean ratio of a variable. Features/genes having higher CV^2 Index is selected from scRNA-seq data. *Fano Factor* is a measure of dispersion among the features. It is also defined as a ratio of variance to mean of a variable. In scRNA-seq data the genes having the highest Fano factor is selected.

Four existing mutual information-based supervised feature selection methods are chosen in our work: Conditional Mutual information maximization (CMIM) [4]- It maximizes the mutual information concerning the class while conditioning upon the selected features, Double Input Symmetrical Relevance (DISR) [5]- It selects a set of variables that can return information on the output class higher than the sum of the information's of each variable taken individually, Joint Mutual Information Maximisation (JMIM) [6]- the 'maximum of the minimum' criterion is used, which alleviates the problem of overestimation of the feature significance, and Minimal-Redundancy-Maximal-Relevance criterion (MRMR)[7]- Feature relevance concerning class labels are considered and ensures that redundant features are not present in the final feature subset [8].

For Gini-Clust, we use the R package with the default parameter as provided in the original paper[1]. For PCA loading, we consider the first three PC components as the default parameter. We use 'praznik' R package with default parameters for supervised methods (MRMR, DISR, JMIM, and CMIM). For *RgCop*, we use regularization coefficient γ as 0.009 (see simulation result on synthetic data). Number of selected features is user defined in our method. In this work, all experiments are performed on top 1000 selected features using *RgCop*.

*Corresponding Author. Email: sumanta.ray@cwi.nl,sanghami@isical.ac.in

2 Description of copula and its related measures

Short description on Copula The 'Copula' term [9] is originated from a Latin word *copulare*, which joins multivariate distributions to its one dimensional distribution function. The copula is considerably employed in high dimensional datasets to obtain joint distributions using uniform marginal distributions and vice versa. According to the famous statistician *Sklar's*, copula function can be defined [10] as:

Copula: A Copula C is an n dimensional function, $C : [0,1]^n \rightarrow [0,1]$, which satisfies the following properties:

1. $C(u_1, \cdots, u_{i-1}, 0, u_{i+1}, \cdots, u_n) = 0$, i.e., the copula is 0 if one of any variable is 0.

2. $C(1, \cdots, 1, u, 1, \cdots, 1) = u$, i.e., the copula function is just u if one of the variable is u with all others being 1.

3. $C(u_1, \cdots, u_n)$ is a non-decreasing function. This implies that, for any hyper rectangle, $R = \{(x_i, y_i) : i \text{ is an integer and } 1 \leq i \leq n\}$, the C volume of R must be non negative, where $(x_i, y_i) \in [0,1]$.

$$V_R(C) = \sum_{i_1=1}^{2} \cdots \sum_{i_n=1}^{2} (-1)^{i_1+\cdots+i_n} C(u_{1,i_1}, \cdots, u_{n,i_n}) \geq 0 \tag{1}$$

   where, $u_{j,1} = x_j$ and $u_{j,2} = y_j$, $j \in (1, \cdots, n)$.

The definition can be properly described by the famous $Sklar's$ theorem.

Sklar's Theorem: Let $X_1, X_2, \cdots, X_n$ be the random vectors whose uniform marginal distributions are $F_1(x_1), F_2(x_2), \cdots, F_n(x_n)$. So, for any joint cumulative distribution function H, their exists a copula function C of its univariate marginal distributions such that,

$$H(x_1, x_2, \cdots, x_n) = C(F_1(x_1), F_2(x_2), \cdots, F_n(x_n)). \tag{2}$$

There are many examples of copulas [11].

One of the extensively used, non-parametric copula family is empirical copula. It is employed to find joint distribution where marginal distributions are unknown. It is defined as follows.

Empirical Copula: Let $X_1, X_2, \cdots, X_n$ be the random variables with marginals cumulative distribution function
$(F_1(x_1), F_2(x_2), \cdots, F_n(x_n))$ respectively.
The empirical estimate of $(F_i, i = 1, \cdots, n)$, based on a sample, $\{x_{i1}, x_{i2}, \cdots, x_{im}\}$ of size m is given by

$$\widehat{F}_i(x) = \frac{1}{m} \sum_{j=1}^{m} 1_{\{X_{ij} \leq x\}}, [i = 1, \cdots, n] \tag{3}$$

The *Empirical Copula* of $X_1, X_2, \cdots, X_n$ is then defined as

$$\begin{aligned} &\widehat{C}(u_1, u_2, \cdots, u_n) \\ &= \frac{1}{m} \sum_{j=1}^{m} 1\{\widehat{F_1}(x_{1,j}) \leq u_1, \widehat{F_2}(x_{2,j}) \leq u_2, \cdots, \widehat{F_n}(x_{n,j}) \leq u_n\}, \end{aligned} \tag{4}$$

for $u_i \in [0,1], [i = 1, \cdots, n]$.
The *Empirical Copula* is employed to model our feature selection method.

Copula correlation measure Let, $Y = \{y_1, y_2\}$ and $Z = \{z_1, z_2\}$ are two bivariate random variables. Their joint distribution function and marginal distributions are
H_{YZ}, $F_Y(y)$ and, $F_Z(z)$ respectively, here, C is their copula distribution function,

$$H_{YZ}(y, z) = C(F_Y(y), F_Z(z)). \tag{5}$$

Kendall tau(τ), the measure of association, [12] can be expressed in terms of concordance and discordance between random variables. Kendall tau is the difference between probability of concordance and discordance of (y_1, y_2) and (z_1, z_2). It can be described as

$$\tau_{YZ} = [P(y_1 - y_2)(z_1 - z_2) \geq 0] - [P(y_1 - y_2)(z_1 - z_2) \leq 0] \tag{6}$$

The relation of Kendall tau measure with copula can be mathematically expressed as:

$$\tau(C_{Y,Z}) = \tau_{YZ} = 4 \iint_{0}^{+1} C(u, v)\, dC(u, v) - 1 \tag{7}$$

Where, $u \in F_Y(y)$ and $v \in F_Z(z)$.
Thus Kendall tau correlation can be described by copula function and is termed as copula-correlation ($Ccor$) in our study.

3 Short description on regularization

Regularization is a type of regression that penalizes the coefficient of redundant feature towards zero xing2001feature (see supplementary for detailed description). The simplest regularization is l_1 norm or Lasso Regression, which adds "absolute value of magnitude" of coefficient as penalty term to the loss function. Another widely used regularization is l_2 norm or Ridge Regression, which adds "squared magnitude" of coefficient as penalty term to the loss function. The key difference between these two is that Lasso shrinks the less important feature's coefficient to zero and thus, removes some features as well. So, this will be applicable where we would have huge number of features. On the contrary, l_1 norm regularization produces sparse solutions by making higher coefficients of the loss function to zero. l_1 norm or Lasso Regression is used in our model to handle the scRNA-seq data with the large number of features. For any vector $A \in \mathcal{R}^m$, the l_1 norm is $||A||_1 = \gamma \sum_{i=1}^{m} |A_i|$, where γ is a tuning parameter, controls penalization. For $\gamma = 0$ regularization effect is none. When γ value increases, it starts to penalizes the larger coefficients to zero. However, after a certain value of γ, the model starts losing important properties, increasing bias in the model and thus causes under-fitting. We tuned γ using eight synthetic Gaussian mixture dataset in this study.

References

[1] Jiang, L., Chen, H., Pinello, L. & Yuan, G.-C. Giniclust: detecting rare cell types from single-cell gene expression data with gini index. *Genome biology* **17**, 144 (2016).

[2] Macosko, E. Z. *et al.* Highly parallel genome-wide expression profiling of individual cells using nanoliter droplets. *Cell* **161**, 1202–1214 (2015).

[3] Grün, D., Kester, L. & Van Oudenaarden, A. Validation of noise models for single-cell transcriptomics. *Nature methods* **11**, 637 (2014).

[4] Fleuret, F. Fast binary feature selection with conditional mutual information. *Journal of Machine Learning Research* **5**, 1531–1555 (2004).

[5] Meyer, P. E. & Bontempi, G. On the use of variable complementarity for feature selection in cancer classification. In *Workshops on Applications of Evolutionary Computation*, 91–102 (Springer, 2006).

[6] Bennasar, M., Hicks, Y. & Setchi, R. Feature selection using joint mutual information maximisation. *Expert Systems with Applications* **42**, 8520–8532 (2015).

[7] Peng, H., Long, F. & Ding, C. Feature selection based on mutual information criteria of max-dependency, max-relevance, and min-redundancy. *IEEE Transactions on pattern analysis and machine intelligence* **27**, 1226–1238 (2005).

[8] Brown, G., Pocock, A., Zhao, M.-J. & Luján, M. Conditional likelihood maximisation: a unifying framework for information theoretic feature selection. *Journal of machine learning research* **13**, 27–66 (2012).

[9] Nelsen, R. B. *An introduction to copulas* (Springer Science & Business Media, 2007).

[10] Jaworski, P., Durante, F., Hardle, W. K. & Rychlik, T. *Copula theory and its applications*, vol. 198 (Springer, 2010).

[11] Durante, F. & Sempi, C. Copula theory: an introduction. In *Copula theory and its applications*, 3–31 (Springer, 2010).

[12] Kruskal, W. H. Ordinal measures of association. *Journal of the American Statistical Association* **53**, 814–861 (1958).
