# Supplementary material for: RgCop-A regularized copula based method for gene selection in single-cell RNA-seq data
Source: PLoS Comput Biol. 2021 Oct 19;17(10):e1009464. doi: 10.1371/journal.pcbi.1009464 (PMC8568278; doi:10.1371/journal.pcbi.1009464)
Supplement: Attachment — Submitted filename: Answer_to_reviewers.pdf [file pcbi.1009464.s003.pdf]

# 1 Answer of Reviewer-1

## 1.1 General Comments

Gene selection is an important step in single cell RNA-seq analysis. The authors proposed a novel regularized copula based method for feature selection. They applied their algorithm on synthetic data and four real scRNA-seq datasets. Their results show an improvement in the performance over the other state-of-the-art methods.

## 1.2 Major Comments

1. The synthetic data was generated using Gaussian distribution. However, scRNA-seq is affected by high dropout rate and the count data is usually very sparse which may follow negative binomial distribution. Now there are some methods for scRNA-seq synthetic data, such as splatter. The authors should try these simulation methods to assess RgCop's performance.

**Answer:** As advised by the reviewer we have now performed the whole simulation experiment with the generated sample from Splatter. We have now replaced the previous experiment (GMM based approach) with the new one. Please see the subsection 'Performance on synthetic scRNA-seq data' (line no.136, page no.4) of the revised version of the manuscript.

2. In figure 3, RgCop was compared with four feature selection techniques. The authors select 10 sets of features ranging from 10 to 100. In most scRNA-seq study, the numbers of genes selected for downstream analysis are rarely under 100. Why do the authors use so few genes? When the selected feature numbers increase to 500, 1000 or 2000, does RgCop still have good performance over other methods? In addition, the stability should be evaluated with more genes.

Answer: As suggested, we have now selected the top 1000 genes to evaluate the downstream analysis and stability of RgCop. The number of selected features are varied in the range from 500 to 1000 to get a set of features as 500,600,700,800,900,1000. The analysis is now updated in the revised version of the manuscript. See figure-2 and figure-3 and subsections' Clustering performance on real dataset using unsupervised method' and 'stability performance' for details.

3. Using genes selected by RgCop, can rare cell populations be identified?

Answer: To see whether the selected genes can discriminate between major and minor subpopulations, we have applied RgCop in two datasets: 1. PBMC68k and 2. 293T and Jurkat cells mixed in vitro in equal proportion [Jindal et al, Discovery of rare cells from voluminous single cell expression data. Nat Commun 9, 4719 (2018)]. We observed that the identified genes have the ability to discover CD14+ monocytes (4.8% of the total cells) and Dendritic subtypes (2.7% of the total cells) from dataset-1 and Jurkat cells (2.5% of the total cells) from dataset 2. This is now described in the subsection 'Rare cell identification' (page no:10, line no.242) of the revised version of the manuscript.

4. In the Relevancy test, genes need to calculate Ccor score with class labels. What does “class label” mean? Is RgCop a supervised method to select genes? In my opinion, Kendall tau is used to measure ordinal association. Are class labels ordered?

Answer: RgCop is a supervised method for gene selection as it selects the initial feature by computing correlation between features and class labels. Here the class labels are the cell types of the original single cell datasets. For defining Kendall tau we used copula, we don’t need to order the labels. Kendall’s tau ($\tau$) is the measure of concordance between two variables; defined as the probability of concordance minus the probability of discordance. The concordance function (Q) is the difference of the probabilities between concordance and discordance between two vectors $(X1, Y1)$ and $(X2, Y2)$ of continuous random variables with different joint distribution $H1$ and $H2$ and common margins $F$ and $G$. It can be proved that the function Q is depend on the distribution of $(X1, Y1)$ and $(X2, Y2)$ only through their copulas. According to Nelson et. al. 2007, there is a relation between Copula and Kendall tau that can be expressed as:

$$\tau = 4E_c[C(U, V)] - 1. \tag{1}$$

where $E_c$ denotes the expectation with respect to the copula distribution function $C$.

5. In the case study of PBMC68k, the ARIs are quite low across all methods. This may be caused by the low gene numbers.

Answer: As suggested, we have now selected the top 1000 genes from PBMC68k. The updated ARI is given in figure 5, panel-E of the revised manuscript.

6. The authors split data in training and test set to prove RgCop’s predicting ability. This result is based on analysis within the same dataset. However, scRNA-seq data may be affected by batch effect. Can RgCop be trained in dataset and predicted accurately in another dataset? Is RgCop influenced by batch effect when it is utilized in a merged data? In addition, it seems RgCop can only predict cells from same tissues.

Answer: As suggested we have now performed an analysis to know how RgCop is influenced by batch effects. For this we have downloaded two datasets from [Tran et al. A benchmark of batch-effect correction methods for single-cell RNA sequencing data (Genome Biol 21, 12 (2020))], consisting of several batches and applied RgCop on it. We have now included a subsection ‘RgCop is robust for data with different batches’ (page no 11, line no. 257) to explain the analysis and the results.

7. How fast is the algorithm? Have you compare the speed between RgCop and other algorithm as the cell number increases?

Answer: We have now performed one analysis to see how RgCop scales with the number of cells (and classes). For this, we have generated simulated data (using splatter) by varying the number of cells (and classes). Four simulated data are generated with the number of cells and classes as follows: 500 cells with two classes, 1000 cells with three classes, 1500 cells with four classes, and 2000 cells with five classes. All data are generated with equal group probabilities, a fixed number of features (2000), dropout rate (0.2), and DE gene proportion (40%). 500 features are selected in each case and the runtime is compared with different competing methods. All experiments were carried out on a Linux server having $50$ cores and $X86\_64$ platform. We have now included the comparisons in the subsection ‘Execution time’ of the revised version of the manuscript. Please see page -11 and -13 and table-6.
Of note, the proposed method is a wrapper-based step wise feature selection method, so it is obvious

that it takes more time than any filter-based feature selection approach (e.g. CV2 Index, Gini Clust). However from the table-5 it can be noticed that the execution time is almost comparable with the other state-of-the-arts.

# 2 Answer of Reviewer 2

## 2.1 General Comments

Informative gene selection is a critical step for scRNA-seq data clustering analysis. In this manuscript, Lall et. al present a new tool called RgCop for informative gene selection from scRNA-seq data. RgCop utilizes copula correlation (Ccor), a robust equitable dependence measure that captures multivariate dependency among a set of genes. The concept of this method is novel. However, some limitations and errors are found during my reading. My concerns are detailed below.

## 2.2 Major comments

1. In the abstract and Fig 1, the description of RgCop give the reader the impression that RgCop can start from unlabeled (unannotated) scRNA-seq dataset to select informative genes. However, in the later part, it seems class labels or in other words, cell annotation is required for RgCop(line 283).What is the point to select genes for a labeled(annotated) dataset? For example, selecting a small number of genes for targeted single-cell RNA-seq for a larger cohort or for probe based spatial transcriptomics. The authors need to clarify what is their goal, otherwise the description is quite confusing.

Ans: RgCop is a supervised gene selection technique and it needs class labels/ cell annotation. However, we can use RgCop for classifying new (unannotated) cell samples. To avoid confusion regarding the applicability of RgCop we have now performed two analyses. First, we show how the selected genes using RgCop are important for discriminating the unknown cell samples. In the second analysis we show how RgCop would be effective on data of several batches. The selected genes from one batch can be applied to cluster the data of another batch of unknown type. Please see the subsection ‘Classifying test samples using the selected feature’ (page no. 08) for the first analysis and subsection ‘RgCop is robust for data with different batches’ (page no. 11) for the second analysis.

2. If selecting minimum marker gene combination is the only goal, I suggest the authors to compare the performance of RgCop with recently polished method called NS-Forest version 2.0(Aevermann, Brian D et.al, Genome Research, 2021). If the authors want to apply RgCop to select genes for clustering unannotated scRNA-seq dataset, then please provide the code and modify the text correspondingly.

Answer: Our goal is to select genes for clustering/discriminating the cells of unknown type. Later we find out cluster specific DE genes which can be treated as markers to annotate the cell clusters. As advised by the reviewer we have now modified the text of the subsection ’marker analysis’ and included two analyses (see subsections ‘Classifying test samples using the selected feature’ and ‘RgCop is robust for data with different batches’) to avoid any confusion about the applicability of the proposed method. The code of the analysis are provided in the github page.

3. Nowadays, it’s normal that the cell number of a single scRNA-seq experiment ranges from $10^4$ to $10^6$. So, scRNA-seq data with a sample size of 68k cannot be regarded as “ultra” large data. It is important to evaluate the performance of RgCop with a dataset with a sample size larger than 500k, or the number of labels larger than 50.

Answer: As suggested by the reviewer, we have included the performance of RgCop in a dataset having sample size larger than 450k. For this, we downloaded mouse single-cell RNA-seq hippocampus dataset from [Saunders, Arpiar, et al. ”Molecular diversity and specializations among the cells of the adult mouse brain.”Cell 174.4 (2018): 1015-1030.] consisting of 690k cells of Adult Mouse Brain and applied RgCop. The analysis is now included in the subsection ‘A case study on ultra large scRNA-seq data’.

## 2.3 Minor comments

4. In the proof of correctness, the authors claim that the gene set $G_s$ obtained from RgCop is optimal. However, there is a error. In line 320, you claim $f(g_k) < f(g_i)$, in fact, $f(g_k) = f(g_i)$ with $k > (i+1)$ is still valid. And $G'_s = G_s$ in this situation. Thus, you cannot claim gene set $G_s$ obtained from RgCop is optimal. Correct me if I am wrong.

Answer: We claim $k > (i+1)$ and for this by the definition of the objective function we can get $f(g_k) < f(g_{i+1})$. In line 387 (in the revised manuscript) we explained why $k$ cannot be equal to $i$, and so $f(g_k) \neq f(g_i)$. If it is then $G'_s$ will contains redundant genes.

5. Please check whether annotations in figures are consistent with corresponding legends. For example, in Fig 3. Panel B, the label of the figures says they are UMAP plots, while legend says they are TSNE plots. More seriously, Fig6. Panel A is a principal component ranking figure, while legend says it is a TSNE plot.

Answer: As advised we have now checked all the legends and corrected the discrepancies.

6. In Fig 3. Panel C, what is the meaning of rows in all heatmaps. Please add an annotation for rows.

Answer: We have now updated the figure-3 (now it is figure-2 in the revised manuscript). The rows represent cells which are annotated by three annotation bar (SC3 clustering ids, cell-type and outlier scores) shown in the top of the heatmap.

7. Why all titles of UMAP in this manuscript is “Leiden”? It’s OK if some UMAP plots show the results of the Leiden clustering method. However, some UMAP plots such as Fig 6.B aren’t designed to show Leiden clusters but showing annotation. Please modify the titles of UMAP plots.

Answer: As advised we have now updated the figures.

8. Please check if legends are consistent with the text. For example, in Fig.3 legend, it says “Each box represents ten ARI scores of clustering results for selected 10 sets of features ranging from 10 to 100”, while in text, it says “We vary the number of selected features in the range from 20 to 100 and compute the ARI scores for each method”. Which one is correct? From 10 to 100 or from 20 to 100?

Answer: As advised we have now corrected the text and the legends.

9. In line 184, it says “Four widely used classifiers” but only three classifiers are listed. It is confusing that the column names of the table2 are ARI and three classifiers. Please make the table2 easy to understand.

Answer: It is now corrected. Please see tble-4 of the revised version.

10. Some contents in text and supplementary are the same. For example, the same pseudo-code of RgCop is presented in both text and supplementary. Please remove the redundancy.

Answer: As advised we have now removed the redundancy.

11. In line 241, it says Yan dataset consists of a transcriptome of 124 individual cells. But table3 shows Yan dataset consists of 90 cells. Which one is correct?

Answer: The raw data consists of 124 individual cells. We downloaded the processed data from https://hemberg-lab.github.io/scRNA.seq.datasets/human/edev/, which consists of 90 cells. This processed data is also utilized in the SC3 clustering work by Kiselev, et al. SC3: consensus clustering of single-cell RNA-seq data. Nat Methods 14, 483–486 (2017). It is now corrected in the main text.

12. In line 247, the GEO accession number is written as "no GSM1832359". Please correct it and other GEO accession numbers.

Answer:It is now corrected

13. A spelling error happened in line 111. Word "doenstream" should be "downstream".

Answer:It is now corrected

# 3 Answer of Reviewer 3

## 3.1 General comments

In this study, the authors developed RgCop, a gene selection method based on regularized copula correlation for scRNA-seq data analysis. RgCop utilizes copula correlation to find the most informative genes from scRNA-seq datasets. In order to filter redundant genes, RgCop employs an l1 regularization term to punish the redundant coefficients of genes. The authors proved that RgCop is superior to other state-of-the-art methods in clustering cells, capturing the dependence between noisy data features, and annotating unknown cells. The main opinions and concerns we raised are listed below:

## 3.2 Major comments

1. The author has provided demo data and RgCop software in Github, which allows users to use the demo data to test RgCop. But the author does not seem to mention how to use RgCop to analyze other scRNA-seq data. In order for the algorithm to be widely used, RgCop needs to allow users to enter their data and adjust parameters.

Answer: As suggested we have now reorganized our Github page with proper documentation. We have also created an R package that can be easily installed using devtools. Please see our Github page for reference.

2. In lines 121 and 122, the authors mentioned "The features are selected by several supervised feature selection algorithm and the classification accuracy are compared with RgCop." What algorithm was applied to select features?

Answer: We compared the performance of RgCop with four widely used supervised feature selection techniques:CMIM, JMIM, DISR , MRMR. It is now updated in the revised version.

3. Based on the Wilcoxon rank-sum test p-value of each cluster, the first five differentially expressed genes are selected as marker genes. These genes will be used to annotate the corresponding clusters. The annotation of each cluster can be directly affected by the marker gene. Therefore, how to select marker genes in scRNA-seq data analysis is a very difficult problem. Why did the authors decide to use the first five genes as marker genes?

Answer: The DE genes are ranked based on their p-value and first five are selected as cluster specific markers. We follow the standard approach of Seurat and scanpy which are well-known pipeline for scRNA-seq analysis. One can use any number of DE genes based on the p-value scores.

4. In line 145, the authors selected 100 characteristic genes to cluster the cells. They then used the Adjusted Rand Index (ARI) to evaluate the clustering performance between the predicted cluster and the known group. Do the top 100 features make RgCop the best performance? Is this number of features consistent in the analysis of different scRNA-seq data? Figure 2A shows that the variation of the ARI value in the Muraro dataset is greater than the Yan and Pollen datasets during the process of changing the number of features from 10 to 100. The Muraro data set contains 2126 instances, which is more than the Yan and Pollen datasets. Does this mean that the size of the feature will significantly affect the performance of RgCop in the datasets with a large number of cells?

Answer: As suggested by the reviewer (and also by other reviewers), we evaluate the performance of RgCop and other competing methods by selecting 1000 genes. We have now updated the clustering results of the competing methods (Figure 2) by varying the number of selected features in the range from 500 to 1000 and compute the ARI scores for each method. There is no optimal number of selected features for RgCop (and for other competing methods as well). The number of selected features is user choice. However, with more number of selected features (here more than 500), ARI performance gets better.
To know the performance of RgCop in data with ultra-large feature size we used mouse single-cell RNA-seq hippocampus dataset from [Saunders, Arpiar, et al. ”Molecular diversity and specializations among the cells of the adult mouse brain.”Cell 174.4 (2018): 1015-1030.] consisting of 690k cells of Adult Mouse Brain. The results shows high ARI score (0.78) which proves the efficacy of RgCop for the dataset with an ultra-large number of cells. Please see subsection ’A case study on ultra large scRNA-seq data’ (page no. 11) of the revised version of the manuscript.

5. In the section “Classification performance on real dataset using supervised method”. Four classification methods were mentioned, but only three were presented. What method was used to measure the accuracy? What is the conclusion of this section?

Answer: We have updated the section by mentioning three classifiers: Neural Network (NNET), Support Vector Machine (SVM), and Gradient Boosting Machine (GBM) to measure the accuracy. . The section represents comparison of RgCop with the benchmark supervised feature selection techniques. The selected features are validated by using three classification techniques. The takeout of this section is that Rgcop outperforms the benchmark for selecting genes for the scrn-seq data. We have now mentioned in the subsection ’classification performance on real dataset using supervised method’, of the revised version of the manuscript.

6. In the abstract, the authors claim that “the differentially expressed (DE) genes identified from the

clusters of scRNA-seq data are found to provide an accurate annotation of cells". However, in the "Marker gene selection" section, the authors only described the differentially expressed genes of each dataset. Based on the result, how to conclude that RgCop can provide an accurate annotation of cells?

Answer: Please note that we follow the standard pipeline/procedure of Seurat/Scanpy for marker gene analysis. The procedure find out the cluster specific DE genes which will be treated as biological markers of the cells within the cluster. To know the annotation of cells using markers (cluster specific DE genes) we have now performed one experiment. We matched the DE genes with experimentally verified cell markers downloaded from CellMarker [Zhang et al., CellMarker: a manually curated resource of cell markers in human and mouse. Nucleic Acids Res. 2019 Jan 8;47(D1):D721-D728.] and annotate the cell clusters with specific type. As we know the labels of cells, we can verify the annotations with the cell labels and observe good annotations (high accuracy). We have now discussed this in the last paragraph of the subsection 'Marker gene selection'

### 3.3 Minor Comments

In the bottom panel of figure 1, the black text with a dark blue background is hard to read.

Answer: The figure-1 is now updated.

Line 110 on page 4: "doenstream" should be "downstream".

Answer: Corrected.
