# Supplementary material for: RgCop-A regularized copula based method for gene selection in single-cell RNA-seq data
Source: PLoS Comput Biol. 2021 Oct 19;17(10):e1009464. doi: 10.1371/journal.pcbi.1009464 (PMC8568278; doi:10.1371/journal.pcbi.1009464)
Supplement: Attachment — Submitted filename: review conmments for RgCop.docx [file pcbi.1009464.s002.docx]

Overall：

Informative gene selection is a critical step for scRNA-seq data clustering analysis. In this manuscript, Lall et. al present a new tool called RgCop for informative gene selection from scRNA-seq data. RgCop utilizes copula correlation (Ccor), a robust equitable dependence measure that captures multivariate dependency among a set of genes. The concept of this method is novel. However, some limitations and errors are found during my reading. My concerns are detailed below.

Major comments:

1. In the abstract and Fig 1, the description of RgCop give the reader the impression that RgCop can start from unlabeled (unannotated) scRNA-seq dataset to select informative genes. However, in the later part, it seems class labels or in other words, cell annotation is required for RgCop(line 283). What is the point to select genes for a labeled(annotated) dataset? For example, selecting a small number of genes for targeted single-cell RNA-seq for a larger cohort or for probe based spatial transcriptomics. The authors need to clarify what is their goal, otherwise the description is quite confusing.

If selecting minimum marker gene combination is the only goal, I suggest the authors to compare the performance of RgCop with recently polished method called NS-Forest version 2.0 (Aevermann, Brian D et.al, Genome Research, 2021).

If the authors want to apply RgCop to select genes for clustering unannotated scRNA-seq dataset, then please provide the code and modify the text correspondingly.

2. Nowadays, it’s normal that the cell number of a single scRNA-seq experiment ranges from${10}^{4}$ to ${10}^{6}$. So, scRNA-seq data with a sample size of 68k cannot be regarded as “ultra” large data. It is important to evaluate the performance of RgCop with a dataset with a sample size larger than 500k, or the number of labels larger than 50.

Minor comments:

1. In the proof of correctness, the authors claim that the gene set$G_{s}$ obtained from RgCop is optimal. However, there is a error. In line 320, you claim $f\left( g_{k} \right)<f(g_{i})$, in fact, $f\left( g_{k} \right)=f(g_{i})$ with $k>(i+1)$ is still valid. And $G_{s'}\neq G_{s}$ in this situation. Thus, you cannot claim gene set$G_{s}$ obtained from RgCop is optimal. Correct me if I am wrong.
2. Please check whether annotations in figures are consistent with corresponding legends. For example, in Fig 3. Panel B, the label of the figures says they are UMAP plots, while legend says they are TSNE plots. More seriously, Fig6. Panel A is a principal component ranking figure, while legend says it is a TSNE plot.
3. In Fig 3. Panel C, what is the meaning of rows in all heatmaps. Please add an annotation for rows.
4. Why all titles of UMAP in this manuscript is “Leiden”? It’s OK if some UMAP plots show the results of the Leiden clustering method. However, some UMAP plots such as Fig 6.B aren’t designed to show Leiden clusters but showing annotation. Please modify the titles of UMAP plots.
5. lease check if legends are consistent with the text. For example, in Fig.3 legend, it says “Each box represents ten ARI scores of clustering results for selected 10 sets of features ranging from 10 to 100”, while in text, it says “We vary the number of selected features in the range from 20 to 100 and compute the ARI scores for each method”. Which one is correct? From 10 to 100 or from 20 to 100?
6. In line 184, it says “Four widely used classifiers” but only three classifiers are listed.

It is confusing that the column names of the table2 are ARI and three classifiers. Please make the table2 easy to understand.

7. Some contents in text and supplementary are the same. For example, the same pseudo-code of RgCop is presented in both text and supplementary. Please remove the redundancy.
8. In line 241, it says Yan dataset consists of a transcriptome of 124 individual cells. But table3 shows Yan dataset consists of 90 cells. Which one is correct?
9. In line 247, the GEO accession number is written as “no GSM1832359”. Please correct it and other GEO accession numbers.
10. A spelling error happened in line 111. Word “doenstream” should be “downstream”.
